# Soil Moisture Content Dominates the Photosynthesis of C_3_ and C_4_ Plants in a Desert Steppe after Long-Term Warming and Increasing Precipitation

**DOI:** 10.3390/plants12162903

**Published:** 2023-08-09

**Authors:** Guangyi Lv, Jing Jin, Mengting He, Chengjie Wang

**Affiliations:** 1 Key Laboratory of Grassland Resources of the Ministry of Education, College of Grassland, Resources and Environment, Inner Mongolia Agricultural University, Hohhot 010018, China; lvguangyi1995@163.com (G.L.); hemengting0325@163.com (M.H.); 2 Mengcao Ecological Environment (Group) Co., Ltd., Inner Mongolia Autonomous Region, Hohhot 010000, China; 13684751295@163.com

**Keywords:** desert steppe, climate change, C_3_ and C_4_ plants, photosynthetic parameters, soil water content

## Abstract

Plant photosynthesis has a non-negligible influence on forage quality and ecosystem carbon sequestration. However, the influence of long-term warming, increasing precipitation, and their interactions on the photosynthesis of dominant species in desert steppe remains unclear, and the main factors regulating plant photosynthesis in desert steppes have remained unrevealed. Therefore, we measured the photosynthetic parameters and specific leaf area of the dominant species and calculated the water and nitrogen content of leaves and soil in a desert steppe after long-term warming and increasing precipitation (air temperature, W0, air temperature increases of 2 °C and 4 °C, W1 and W2; natural precipitation, P0, natural precipitation increases of 25% and 50%, P1 and P2). Results showed that warming and increasing precipitation significantly enhanced photosynthesis in C_3_ and C_4_ species (*p* < 0.05). Compared to W0P0, the net photosynthetic rate of C_3_ and C_4_ species in W2P2 increased by 159.46% and 178.88%, respectively. Redundancy analysis showed that soil water content significantly explained the photosynthesis of C_3_ and C_4_ plants (the degree of explanation was 48% and 67.7%), followed by soil-available nitrogen content (the degree of explanation was 19.6% and 5.3%). Therefore, our study found that climate change enhanced photosynthesis in C_3_ and C_4_ plants, and soil water content plays a critical role in regulating photosynthesis in desert steppes.

## 1. Introduction

Photosynthesis, as a crucial physiological process in plants, can convert light energy into biochemical energy and involves the absorption of carbon dioxide and the release of oxygen [1], which not only dominates ecosystem carbon exchange but also has an essential impact on food security [2,3,4]. Depending on the differences in the photosynthetic pathway, plants were classified into three types, i.e., Crassulacean Acid Metabolism (CAM), C_3_ and C_4_ species [5], which resulted in their contrasting responses to climate change [6,7]. For example, C_3_ species are attracted to lower temperatures and wetter environmental conditions, while C_4_ plants are better suited to warm and dry environmental conditions [6,8]. In addition to temperature and precipitation, the use efficiency of light, nitrogen, water, and carbon dioxide have a significant influence on the photosynthesis of C_3_ and C_4_ plants [9,10,11,12,13]. Therefore, photosynthesis in C_3_ and C_4_ plants is regulated by multiple factors.

Due to physiological differences between C_3_ and C_4_ species, Ehleringer proposed the Quantum Yield Hypothesis in 1978 [9]. His hypothesis was that temperature plays an important role in the photosynthesis of C_3_ and C_4_ species. In the same year, Hatch considered that C_4_ species possess a special leaf structure that allows C_4_ plants to have higher photosynthesis than C_3_ plants in dry and warm conditions [13]. Therefore, a common phenomenon is that higher temperatures have a positive effect on the photosynthesis of C_4_ species and a negative effect on the photosynthesis of C_3_ species [6,9]. In addition, the nitrogen use efficiency of C_3_ and C_4_ plants significantly correlated with their photosynthesis. Brown believed that the nitrogen use efficiency of C_4_ plants was higher than C_3_ plants [10], which means that C_4_ species can sustain higher photosynthesis at lower leaf nitrogen levels. In controlled indoor experiments, Sage and Pearcy confirmed this hypothesis [14]. They concluded that the photosynthetic rate of C_4_ plants was stronger than C_3_ plants at all treatments of leaf nitrogen content. The special leaf physiological structure of C_4_ plants causes higher nitrogen use efficiency, which is regarded as being affected by phylogenetic control or climate change [15,16]. Equally essential as nitrogen use efficiency is the water use efficiency of C_3_ and C_4_ plants. Winslow et al. pioneered the water availability hypothesis [11], and they concluded that the photosynthetic rates of C_3_ and C_4_ species were affected by water availability. Niu et al. confirmed this hypothesis using indoor controlled experiments [17]. They deemed that water availability plays a crucial role in plant photosynthesis. Severe soil water deficiency inhibited photosynthesis in C_3_ and C_4_ plants and negatively affected forage quality [18,19,20]. In summary, photosynthesis is influenced by a variety of factors, all of which may be altered by warming and increased precipitation.

The fifth report of the IPCC pointed out that climate warming and frequent precipitation will be shifted to mid and high latitudes in the northern hemisphere [21], which may alter the photosynthesis of C_3_ and C_4_ plants, intensify interspecific competition for species and increase the uncertainty of ecosystems [22,23,24]. Earlier studies have shown that warming and increased precipitation improved photosynthesis in C_3_ and C_4_ species [25,26]. A temperature threshold existed for the influence of warming on photosynthesis in C_3_ and C_4_ plants. When ambient temperatures fall below the threshold, warming increases photosynthesis and conversely inhibits photosynthesis [27]. The temperature threshold varies with species, in which the optimum temperature for photosynthesis was higher in C_4_ species than in C_3_ species [25,28]. In addition, precipitation has a significant effect on the temperature threshold. The temperature threshold gradually increased with increasing precipitation [29], which means that the interaction of warming and increased precipitation may significantly improve photosynthesis in C_3_ and C_4_ plants [26,30]. However, Song et al. [28] showed that the interaction of warming and increasing precipitation had an insignificant impact on plant photosynthesis in the temperate grasslands of Inner Mongolia. Thus, the influence of warming and increasing precipitation on plant photosynthesis may vary with grassland types.

The desert steppe is situated in the east of the Eurasian temperate steppe. It has much less vegetation and is vulnerable to desertification [31]. Although the impact of warming or increased precipitation on the photosynthesis of dominant species in desert steppes has been studied [29], their interaction has rarely been reported. In addition, the main factor regulating photosynthesis of dominant species in desert steppes after long-term warming and increasing precipitation has remained unrevealed. Therefore, we measured the photosynthetic parameters and specific leaf area of the dominant species, calculated the water and nitrogen content of leaves and soil in a desert steppe after eight years of warming and increasing precipitation, and made the following hypotheses: (1) Warming and increasing precipitation will improve photosynthesis in the C_3_ and C_4_ species of a desert steppe. (2) Based on the quantum yield hypothesis, we consider that temperature has a significant effect on the photosynthesis of dominant species in a desert steppe.

## 2. Results

### 2.1. Photosynthesis of C_3_ and C_4_ Plants

**Figure 1:**
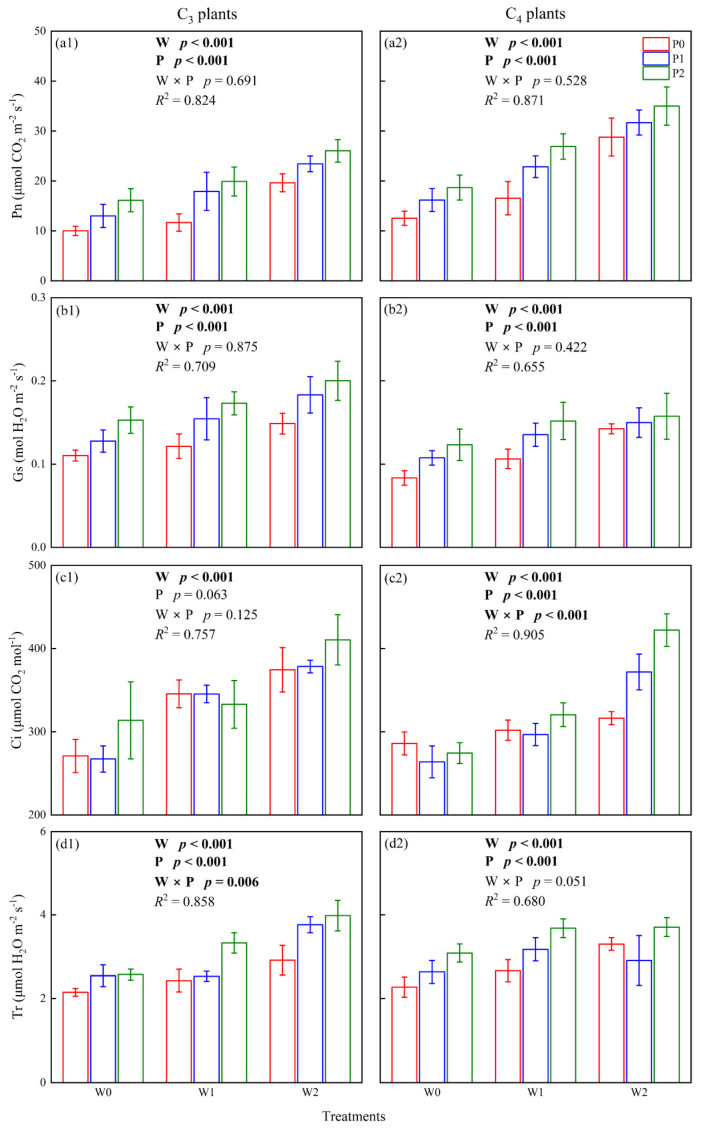
The effects of climate warming (W), increased precipitation (P), and their interaction (W × P) on the (**a1**,**a2**): Pn (net photosynthetic rate), (**b1**,**b2**): Gs (stomatal conductance), (**c1**,**c2**): Ci (intercellular carbon dioxide), and (**d1**,**d2**) Tr (transpiration rate). W0, W1, and W2 are near-surface air temperatures, near-surface air temperature increased by 2 °C and 4 °C. P0, P1, and P2 are ambient precipitation, ambient precipitation increased by 25% and 50%. Same as below.

Climate warming and increased precipitation had a significant impact on the net photosynthetic rate (Pn), stomatal conductance (Gs), and transpiration rate (Tr) in C_3_ and C_4_ species (*p* < 0.05, Figure 1a,b,d), while the effect of increased precipitation on the intercellular carbon dioxide (Ci) of C_3_ plants was insignificant (*p* > 0.05, Figure 1c). In addition, the interaction of warming and increasing precipitation had a significant impact on the Ci in C_4_ plants and the Tr in C_3_ plants (*p* < 0.05, Figure 1c,d). The one-way ANOVA results showed that warming and increasing precipitation significantly improved the Pn, Gs, Ci, and Tr of C_3_ and C_4_ plants (*p* < 0.05, Figure 1). Specifically, the Pn, Gs, Ci, and Tr of C_3_ plants in W0P0 and W2P2 were 10.04 ± 0.92 and 26.05 ± 2.26 µmol CO_2_ m^−2^ s^−1^, 0.11 ± 0.01 and 0.20 ± 0.02 mol H_2_O m^−2^ s^−1^, 270.99 ± 19.96 and 410.60 ± 30.32 µmol CO_2_ mol^−1^, 2.15 ± 0.09 and 3.98 ± 0.37 µmol H_2_O m^−2^ s^−1^. The Pn, Gs, Ci, and Tr of C_4_ plants in W0P0 and W2P2 were 12.55 ± 1.42 and 35.00 ± 3.83 µmol CO_2_ m^−2^ s^−1^, 0.08 ± 0.01 and 0.16 ± 0.03 mol H_2_O m^−2^ s^−1^, 286.13 ± 13.76 and 422.26 ± 19.61 µmol CO_2_ mol^−1^, 2.27 ± 0.24 and 3.71 ± 0.23 µmol H_2_O m^−2^ s^−1^, respectively (Figure 1).

### 2.2. Functional Traits of C_3_ and C_4_ Leaves

**Figure 2:**
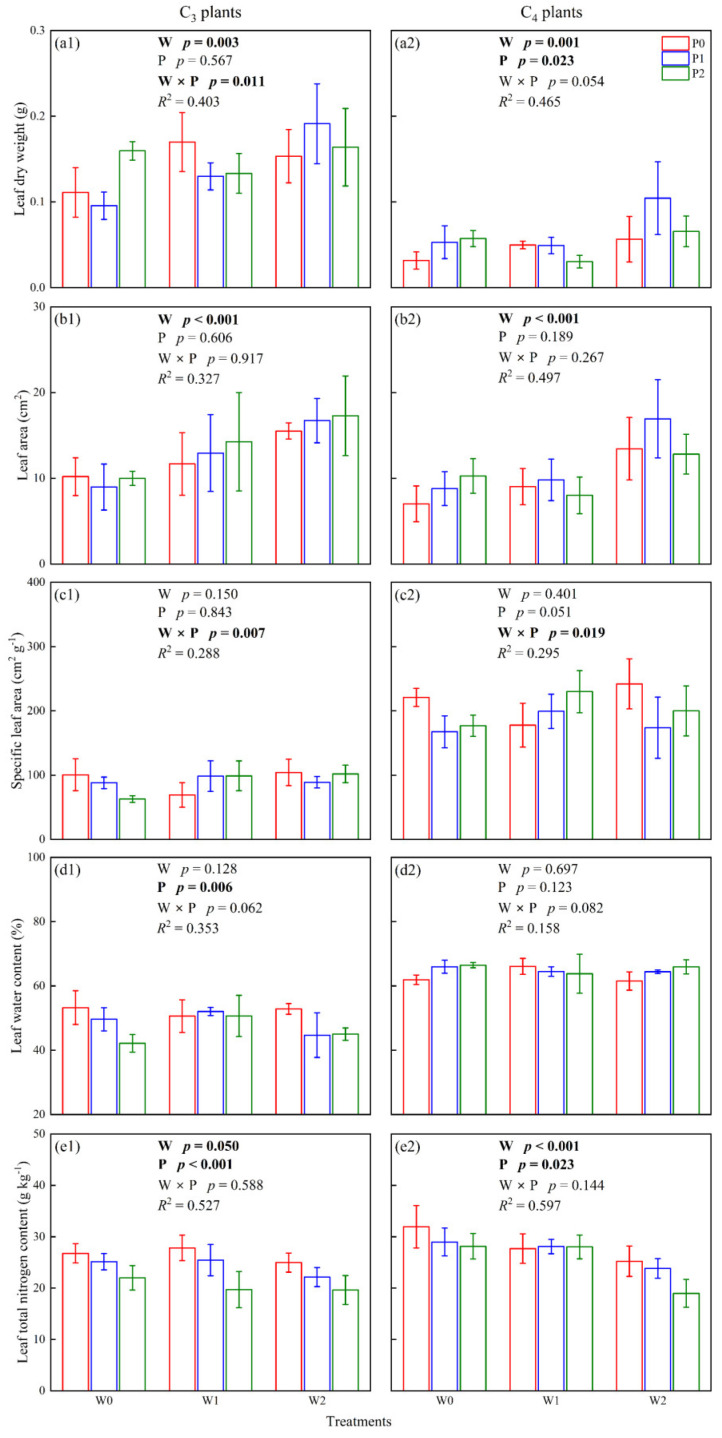
The effects of climate warming (W), increased precipitation (P), and their interaction (W × P) on the leaf dry weight (**a1**,**a2**), leaf area (**b1**,**b2**), specific leaf area (**c1**,**c2**), leaf water content (**d1**,**d2**) and leaf total nitrogen content (**e1**,**e2**) of C_3_ and C_4_ leaves.

The dry weight and total nitrogen of leaves were mainly affected by warming and increased precipitation (*p* < 0.05, Figure 2a,e), while the leaf area was mainly influenced by warming and the specific leaf area was mainly influenced by the interaction of warming and increasing precipitation (*p* < 0.05, Figure 2b,c). The one-way ANOVA results showed that warming and increased precipitation significantly increased the leaf dry weight and area of C_3_ and C_4_ species (*p* < 0.05, Figure 2), in which the leaf dry weight and area of C_3_ plants in W0P0 and W2P2 were 0.11 ± 0.03 and 0.16 ± 0.05 g, 10.19 ± 2.21 and 17.29 ± 4.65 cm^2^, respectively. The leaf dry weight and area of C_4_ plants in W0P0 and W2P2 were 0.03 ± 0.01 and 0.07 ± 0.02 g, 7.01 ± 2.09 and 12.81 ± 2.33 cm^2^, respectively. In addition, warming and increased precipitation significantly reduced the leaf nitrogen content of C_3_ and C_4_ species (*p* < 0.05, Figure 2), in which the leaf nitrogen content of C_3_ plants in W0P0 and W2P2 were 26.78 ± 1.86 and 19.63 ± 2.81 g kg^−1^, and the leaf nitrogen content of C_4_ plants in W0P0 and W2P2 were 31.95 ± 4.12 and 18.98 ± 2.71 g kg^−1^.

### 2.3. Soil Physicochemical Parameters

**Figure 3:**
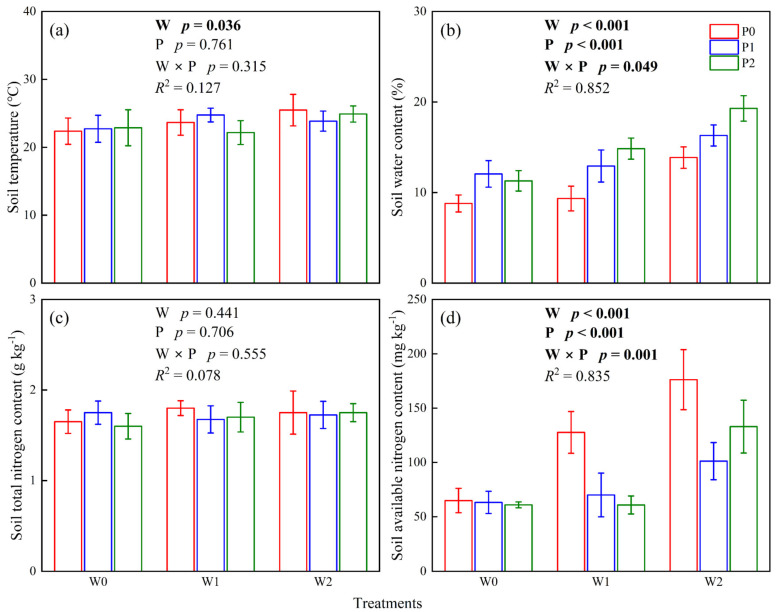
The effects of climate warming (W), increased precipitation (P), and their interaction (W×P) on the soil temperature (**a**), soil water content (**b**), soil total nitrogen content (**c**) and soil available nitrogen content (**d**).

Results showed that warming, increasing precipitation, and their interactions significantly affected soil water content and soil-available nitrogen content (*p* < 0.05, Figure 3b,d), while insignificantly affected soil temperature and soil total nitrogen content (*p* > 0.05, Figure 3a,c). The one-way ANOVA results showed that warming and increasing precipitation significantly increased soil water content and soil-available nitrogen content (*p* < 0.05, Figure 3b,d), in which the soil water content in W0P0 and W2P2 were 8.79 ± 0.94 and 19.30 ± 1.41%, and the soil-available nitrogen content in W0P0 and W2P2 were 64.93 ± 11.25 and 133.01 ± 24.30 mg kg^−1^ (Figure 3b,d).

### 2.4. Redundancy Analysis

**Figure 4:**
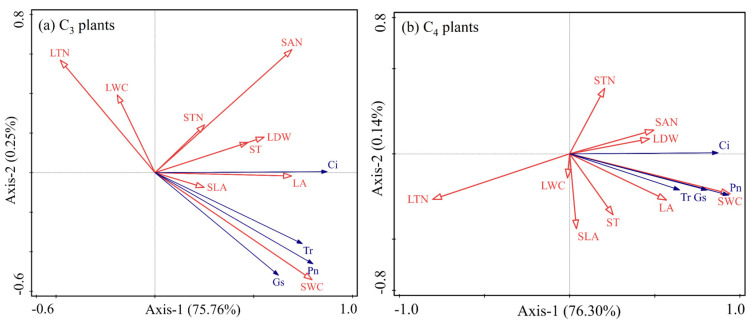
Redundancy analysis of photosynthesis in C_3_ (**a**) and C_4_ (**b**) plants and environmental factors. Pn, Gs, Ci, and Tr were net photosynthetic rate, stomatal conductance, intercellular carbon dioxide, and transpiration rate, respectively. LDW, LA, SLA, LWC, and LTN were leaf dry weight, leaf area, specific leaf area, leaf water content, and leaf total nitrogen content, respectively. ST, SWC, STN, and SAN were soil temperature, soil water content, soil total nitrogen content, and soil-available nitrogen content, respectively.

Redundancy analysis showed that the photosynthesis parameters of C_3_ plants were positively correlated with their leaf area, leaf dry weight, and soil water content, while being negatively correlated with leaf water content and leaf nitrogen content (Figure 4a). The photosynthesis parameters of C_4_ plants were positively correlated with their leaf area, leaf dry weight, soil water content, and soil-available nitrogen content, while being negatively correlated with their leaf nitrogen content (Figure 4b). Additionally, soil water content significantly explained the photosynthesis of C_3_ and C_4_ species, followed by soil-available nitrogen content and leaf total nitrogen content (*p* < 0.01, Table 1). The other factors insignificantly explained the photosynthesis of C_3_ and C_4_ plants (*p* > 0.01, Table 1).

## 3. Discussion

Climate warming and extreme precipitation events are shifting to the middle and high latitudes of the Northern Hemisphere [21], which is severely affecting the ecophysiological functions of vegetation and increasing the uncertainty of grassland ecosystems [23,32]. Differences in the photosynthetic pathways of C_3_ and C_4_ plants result in their contrasting responses to climate change, which means that the competition for photosynthetic resources between C_3_ and C_4_ species intensifies with climate change [33]. Therefore, we measured photosynthetic parameters, water, and nitrogen content in leaves of C_3_ and C_4_ species in the desert steppe after warming and increased precipitation, explored the changing trends in photosynthesis, and revealed the main factors regulating photosynthesis in C_3_ and C_4_ species of a desert steppe.

Earlier studies have shown that the use efficiency of light, nitrogen, and water dominates the photosynthesis and interspecific competition of C_3_ and C_4_ species in grasslands [9,10,11], in which the Quantum Yield Hypothesis is widely accepted. A common phenomenon is that climate warming improves photosynthesis in C_4_ species and inhibits photosynthesis in C_3_ species [34,35,36]. On the one hand, increased ambient temperature closes leaf stomata and inhibits photosynthesis of C_3_ species [29]. In contrast, the smaller intercellular CO_2_ concentration required for photosynthesis in C_4_ species means that lower levels of stomatal conductance and transpiration rates have an insignificant influence on photosynthesis in C_4_ species [37]. On the other hand, C_4_ species have unique leaf anatomy (Kranz anatomy), which enables C_4_ species to maintain a high level of photosynthesis at high temperatures [8,13]. However, warming and increasing precipitation significantly promoted photosynthesis in C_3_ and C_4_ plants in our study (Figure 1). We suggested the following reasons for the enhanced photosynthesis of C_3_ and C_4_ plants: (1) Warming and increased precipitation significantly increased the leaf area of C_3_ and C_4_ species in our study (Figure 2), which is considered to be the main factor in improving photosynthesis. (2) The mean air temperature during the plant growing season was 15.41 °C (Figure S1), which was not sufficient to suppress photosynthesis in C_3_ species. Collatz et al. [38] believed that C_3_ species will lose its competitive advantage in monthly mean temperatures above 22 °C. (3) Increasing precipitation improved the stomatal conductance of C_3_ leaves, which benefits photosynthesis in C_3_ plants (Figure 1). The optimum temperature for photosynthesis in C_3_ plants is raised with increasing soil water content [29]. (4) The C_3_ species selected for this experiment was *S. breviflora*, which is a constructive species in desert steppes and has a strong ability to adapt to climate change. Therefore, warming and increasing precipitation will not inhibit the photosynthesis of C_3_ species in a desert steppe. This was consistent with the research by Niu et al. [34], who considered that most species in the grasslands of northern China were adapted to climate change.

Changes in plant or soil nitrogen content have a significant influence on the photosynthesis of C_3_ and C_4_ plants. A high photosynthetic rate corresponds to high leaf nitrogen content [33,39]. Although the intrinsic leaf nitrogen content of C_4_ species was lower than C_3_ plants [16], C_4_ plants have higher nitrogen use efficiency than C_3_ plants, which means the total nitrogen content per unit area of leaves in C_4_ species can fix more CO_2_ [10]. Yuan et al. [40] concluded that leaf nitrogen content is regulated by soil-available nitrogen content. The photosynthetic rate of C_4_ species was consistently higher than that of C_3_ species at any given nitrogen concentration, independent of interspecific competition. However, warming and increasing precipitation reduced leaf nitrogen content in selected species (Figure 2), and the leaf nitrogen content was negatively correlated with photosynthesis in our study (Figure 4). It may be that warming and increasing precipitation improved the soil availability of nitrogen content (Figure 3), which benefits the growth of perennial forbs and annual herbs, resulting in lower nitrogen uptake efficiency by perennial grasses. This is consistent with the findings of Yang et al., who considered that warming and increased precipitation reduced the proportion of perennial grasses in the plant community [41]. In addition, Tian et al. [42] also considered that perennial forbs preferred to grow in higher soil nitrogen content.

Water has a crucial influence on species composition and distribution in plant communities in arid grassland ecosystems. Soil water availability not only affected the photosynthesis of C_3_ and C_4_ plants but also determined C_3_/C_4_ in plant communities of grassland [11]. Under severe drought conditions, leaves curl up and close their stomata, inhibiting plant photosynthesis [8]. On the contrary, higher soil moisture content enables plants to stretch their leaves and improves their photosynthesis and water use efficiency [42]. This is consistent with the results of our study. Warming and increased precipitation improved photosynthesis in C_3_ and C_4_ plants (Figure 1). Ghannoum et al. [43] suggested that the influence of water stress on photosynthesis in C_4_ species mainly depended on biochemical limitations. As research progressed, he changed this view and concluded that the inhibition of photosynthesis by water stress was limited by stomata in the early stages, and by non-stomata in the later stages, C_4_ species were more sensitive to water stress than C_3_ species [37]. Recent studies have shown that long-term warming altered the leaf structure of C_3_ and C_4_ plants, which depended on soil water content [22]. In our study, soil water content significantly explained the photosynthesis of C_3_ and C_4_ plants, in which soil moisture content explained 48.0% of photosynthesis in C_3_ plants and 67.7% of photosynthesis in C_4_ plants (Table 1). Therefore, we suggested that soil water content plays a vital role in regulating photosynthesis in C_3_ and C_4_ species of desert steppes under warming and increased precipitation. This was consistent with previous studies, which have considered that soil water availability dominated species composition and grassland productivity in Inner Mongolian grasslands [41].

## 4. Materials and Methods

### 4.1. Study Site

The study area is located in Siziwang Banner (41°47′20″ N, 111°53′46″ E), Inner Mongolia, northern China. The mean temperature of the study site in the plant-growing season was 15.41 °C (Figure S1a), and the total precipitation of the study site in the plant-growing season was about 236.76 mm (Figure S1b). According to the FAO soils classification system, the soil in the study site belongs to Haplic Calcisols. The species in the study site are mainly *Artemisia frigida* Willd., *Stipa breviflora* Griseb., *Cleistogenes songorica* Roshev., and *Kochia prostrata* (L.) Schrad. Table S1 shows the composition and classification of the species in our study site [44,45].

### 4.2. Experimental Design

**Figure 5:**
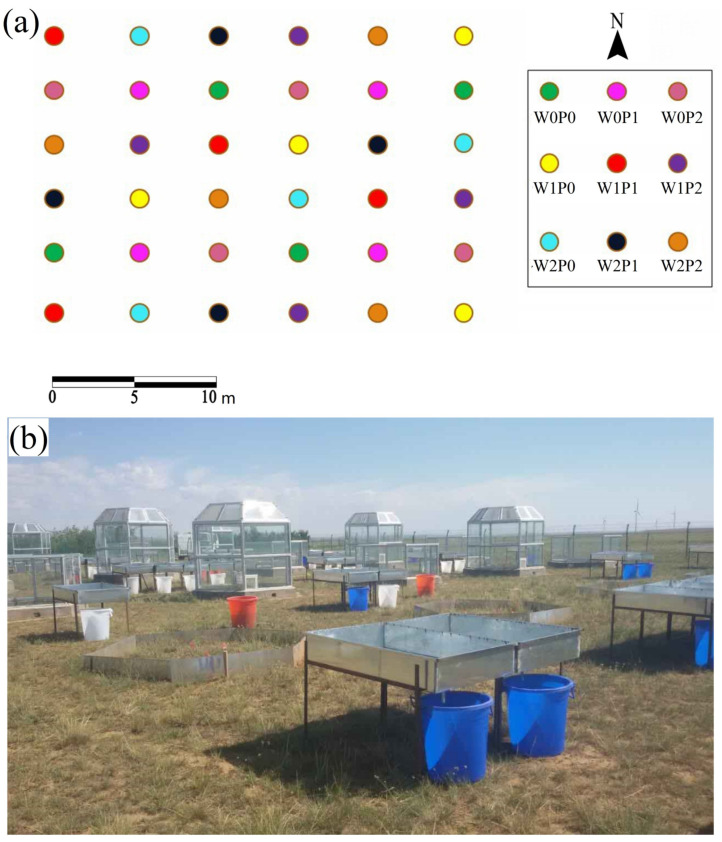
Study site (**a**) and the equipment for warming and increased precipitation (**b**). W0, W1, and W2 are near-surface air temperatures, near-surface air temperature increased by 2 °C and 4 °C. P0, P1, and P2 are ambient precipitation, ambient precipitation increased by 25% and 50%.

The Inner Mongolia Grassland Scientific Research Centre set up a factorial experiment on warming and increased precipitation in 2014. Temperature and precipitation during the plant growing season were the controlling factors for the experiment, with three levels for each factor, i.e., air temperature (W0), air temperature increase of 2 °C and 4 °C (W1 and W2), natural precipitation (P0), natural precipitation increase of 25% and 50% (P1 and P2). The equipment used to simulate warming is an open-top chamber (OTC). The bottom of OTC is a regular hexagon, and the side length was 1.5 m. The heights of the OTCs are 1 m (W1) and 2.3 m (W2), respectively (Figure 5b). The light transmission of the OTC is over 95%. It has two fans for air circulation. Additionally, the rain collector was used to collect natural precipitation (Figure 5b). Their area was 25% and 50% of the OTC base area, respectively. During the plant growing season, they collected natural rainfall in designated buckets, then the worker artificially spread the collected precipitation in each OTC in order to obtain a higher level of precipitation. The experiment was designed in a randomized block group with nine treatments, and each treatment had four replicates, totaling 36 plots (Figure 5a).

### 4.3. Plant and Soil Sampling

Experimental samples were collected in mid-August 2022. Firstly, according to previous studies on dominant species in our study area, we selected *S. breviflora* (C_3_ species) and *C. songorica* (C_4_ species) to represent changes in photosynthesis, nitrogen, and water content in C_3_ and C_4_ species in the desert steppe under long-term warming and increasing precipitation. The selected species are perennial grasses, which make up a high proportion of the community in desert steppes [44,45]. Secondly, we used the open system infrared gas analyzer with a leaf chamber (LI-6400XT, LI-COR, Lincoln, NE, USA) to measure the photosynthesis of C_3_ and C_4_ leaves. On a sunny morning from eight to eleven o’clock, the leaves of our selected species covered the chamber, and the net photosynthetic rate, transpiration rate, stomatal conductance, and intercellular carbon dioxide concentration of the leaves were measured. Three to five measurements were taken per species and repeated for three days. To measure the leaf fresh weight, leaf area, and leaf dry weight of selected species, the leaves were cut off after measuring photosynthesis. The specific leaf area and leaf water content of each species were calculated from the fresh weight, area, and dry weight of the leaves. Additionally, we measured the leaf nitrogen content of each species by using an elemental analyzer (Vario Isotope Select, Elementar, Germany). Finally, we collected surface soil (0–10 cm) from each OTC using an aluminum box and measured soil moisture content, soil total, and available nitrogen content.

### 4.4. Statistical Analysis

Prior to data analysis, the Shapiro–Wilk test was carried out in IBM SPSS Statistics 26 (Armonk, NY, USA) to check the data in this study for normal distribution, and all data passed the test. We used the general linear model to analyze the effects of warming (W), increased precipitation (P), and their interactions (W × P) on C_3_ and C_4_ plants and soil. One-way ANOVA was performed to analyze the differences in C_3_ and C_4_ plants and soil under warming and increasing precipitation. The general linear model is as follows:

(1) Y=W+P+W×P+I

where *Y* is an index of plant or soil, *W* is warming, *P* is increased precipitation, × is interaction, and I is the intercept.

In addition, Redundancy analysis (RDA) was carried out in Canoco 5, which was used to reveal the relationship between environmental factors and plant photosynthesis. In this paper, we used Microsoft Excel 2019 to make tables and used Origin Pro 2023b (Origin Lab, Northampton, MA, USA) to make figures.

## 5. Conclusions

Warming and increasing precipitation have a crucial impact on the photosynthesis of dominant species in desert steppes. We found that long-term climate warming and increasing precipitation significantly improved photosynthesis in C_3_ and C_4_ species, and the dominant species in desert steppes have adapted to climate change, which confirmed our first hypothesis. In addition, soil water content significantly explained the photosynthesis of C_3_ and C_4_ plants in our study, and soil water content plays an essential role in regulating photosynthesis in desert steppes rather than temperature. Therefore, our study revealed the changing trends of photosynthesis in dominant species of desert a steppe after warming and increased precipitation and its regulating factors, which provided a theoretical basis for predicting carbon sequestration in desert steppes under climate change.

## Figures and Tables

**Table 1:** Explanation of environmental factors on photosynthesis in C_3_ and C_4_ plants. LDW, LA, SLA, LWC, and LTN were leaf dry weight, leaf area, specific leaf area, leaf water content, and leaf total nitrogen content, respectively. ST, SWC, STN, and SAN were soil temperature, soil water content, soil total nitrogen content, and soil-available nitrogen content, respectively. * and ** were significant correlations at *p* < 0.05 and *p* < 0.01.

Photosynthesis in C_3_ Plants					Photosynthesis in C_4_ Plants			
Factors	Explains %	F	*p*		Factors	Explains %	F	*p*
SWC	48.0	31.4	0.002 **		SWC	67.7	71.2	0.001 **
SAN	19.6	20.0	0.002 **		SAN	5.3	6.5	0.024 *
LTN	3.6	4.1	0.050 *		LTN	1.4	1.8	0.220
LDW	1.6	1.8	0.184		STN	0.5	0.6	0.466
ST	2.5	3.0	0.094		LWC	0.4	0.5	0.528
SLA	0.3	0.3	0.572		LDW	0.3	0.4	0.520
STN	0.2	0.3	0.636		ST	0.4	0.4	0.528
LA	0.1	0.1	0.696		LA	0.4	0.4	0.550
LWC	<0.1	<0.1	0.756		SLA	<0.1	<0.1	0.786

## Data Availability

The data that support the findings of this study are available from the corresponding author upon reasonable request.

## Supplementary Materials

The following supporting information can be downloaded at: https://www.mdpi.com/article/10.3390/plants12162903/s1, Table S1: Species composition and division of plant communities in study site; Figure S1: Air temperature (a) and natural precipitation (b) of the plant growing season (May to October) of 2022.
