# Supplementary material for: Soil Moisture Content Dominates the Photosynthesis of C3 and C4 Plants in a Desert Steppe after Long-Term Warming and Increasing Precipitation
Source: Plants (Basel). 2023 Aug 9;12(16):2903. doi: 10.3390/plants12162903 (PMC10459209; doi:10.3390/plants12162903)
Supplement: Supplementary file 1 [file plants-12-02903-s001.zip › plants-2461893-supplementary.pdf]

plants

Supplementary material

Table S1. Species composition and division of plant communities in study site.

Species Name	Functional Group	Photosynthesis Type
Stipa breviflora Griseb.	PG	C_3
Stipa krylovii Roshev.	PG	C_3
Leymus chinensis (Trin.) Tzvel.	PG	C_3
Cleistogenes songorica (Roshev.) Ohwi.	PG	C_4
Allium mongolicum Regel.	PF	C_3
Convolvulus ammannii Desr.	PF	C_3
Potentilla bifurca L.	PF	C_3
Aster altaicus Willd.	PF	C_3
Iris tectorum Maxim.	PF	C_3
Astragalus galactites Pall.	PF	C_3
Melissilus ruthenicus (L.) Peschkova.	PF	C_3
Allium polyrhizum Turcz. ex Regel.	PF	C_3
Artemisia frigida Willd.	SS	C_3
Kochia prostrata (L.) Schrad.	SS	C_4
Caragana microphylla Lam.	S	C_3
Caragana stenophylla Pojark.	S	C_3
Neopallasia pectinata (Palls) Poljak.	AH	C_3
Artemisia scoparia Waldst. et Kit.	AH	C_3
Chenopodium glaucum L.	AH	C_4
Chenopodium aristatum L.	AH	C_4
Portulaca oleracea L.	AH	C_4
Salsola collina Pall.	AH	C_4

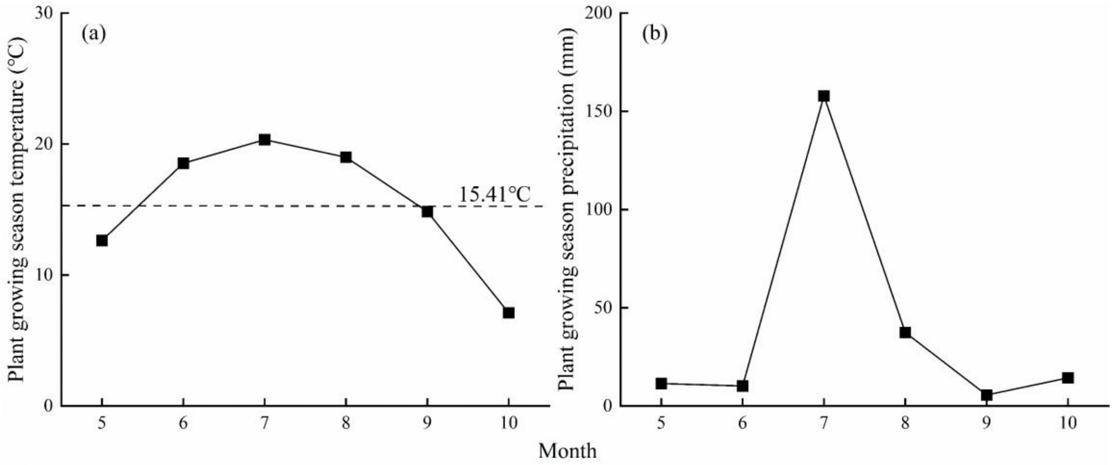

Figure S1. Air temperature (a) and natural precipitation (b) of the plant growing season (May to October) of 2022.
